# Unmet needs in allergic rhinitis: international survey on management of allergic rhinitis by physician and patient: the optimal management (ISMAR 2 study)

**DOI:** 10.1186/1939-4551-8-S1-A239

**Published:** 2015-04-08

**Authors:** Carlos E Baena-Cagnani, Ashok Mahashur, Jamal Jawad, Margarita Murrieta-Aguttes, Faheem A Tadros, Mohammad Gharagozlou, Talha Mahmud

**Affiliations:** 1 Research Centre in Respiratory Medicine, Argentina; 2 Hinduja Hospital, India; 3 Dr Soliman Fakeeh Hospital, Saudi Arabia; 4 Sanofi, France; 5 Al Zahra Hospital, United Arab Emirates; 6 Children's Medical Center, Iran; 7 Pakistan

## Background

Allergic Rhinitis (AR) is a worldwide spread disease and has an important impact on social life, sleep quality (SQ), school and work productivity and huge costs. ISMAR was designed to identify attitudes and trends among physicians managing AR in different parts of the world. ISMAR 2 is the second phase.

## Methods

ISMAR is an international, multicenter, non-interventional, cross-sectional study conducted in adults and children (≥ 6 years) with physician diagnosis-AR of at least 1 year of duration. Physicians recruited consecutive patients to whom the ISMAR questionnaire was administered. Data collection was performed during a single visit. Other 2 additional documents (the investigator's questionnaire and Case Record Form) were also filled in. Statistical analysis was descriptive. For statistical inferences, Student tor Wilcoxon test were performed according to the variables normality. Herein we show the results on Patient Optimal Management.

Patients fulfilling the following criteria were considered as optimally managed:

- Patient education, environmental control and allergen avoidance

- Optimal pharmacological treatment according to ARIA guidelines

## Results

Two thousand three hundred patients (2298 were analyzed), mean age 28.8 ± 15.9 years, 54.4% of males were included in 5 new countries and in 4 countries (new centres) that already participated in ISMAR phase 1. The nasal symptoms frequency was < 4 days per week (29.5%), < 4 consecutive weeks (19.5%), > 4 days per week (29.1%) and > 4 consecutive weeks (21.8%) [N=2207]. 85.7% of patients had never smoked. Current smokers suffered most commonly of persistent rhinitis (61.3%). Patients reported having within the past 12 months, most frequently: recurrent cough (57.1%), nocturnal cough (40.8%), recurrent dyspnea/breathlessness (36.5%), dyspnea/breathlessness after exercise (33.1%), recurrent wheezing (32.1%), cough after exercise (29.8%), nocturnal dyspnea/breathlessness (29.5%). AR symptoms impaired sleep (63.5%), mood (61.9%), physical activities (50.4%), and working performance (42.1%). 96.3% of patients received recommendations to avoid allergens and irritants. 96.6% were receiving AR treatment, mainly antihistamines and nasal corticosteroids but also anti-leukotrienes and nasal and oral decongestants. 55.8% of patients had an optimal treatment. Patients with a frequency of nasal symptoms > 4 days per week and > 4 consecutive weeks and those with recurrent dyspnea/breathlessness appeared as more frequently optimally managed (p<0.001 and P<0.018, respectively).

## Conclusions

This study shows that optimal management in terms of fulfilling the management recommendations is observed in only 55.8% of patients. The application of ARIA guidelines could be improved in certain countries.

